# Accelerated Coronary Angiogenesis by *Vegfr1*-Knockout Endocardial Cells

**DOI:** 10.1371/journal.pone.0070570

**Published:** 2013-07-24

**Authors:** Zheng Zhang, Bin Zhou

**Affiliations:** 1 The State Key Laboratory of Biotherapy, West China Medical School of Sichuan University, Chengdu, Sichuan, China; 2 Departments of Genetics, Albert Einstein College of Medicine, Bronx, New York, United States of America; 3 Departments of Pediatrics and Medicine (Cardiology), Wilf Family Cardiovascular Research Institute, Albert Einstein College of Medicine, Bronx, New York, United States of America; 4 Department of Cardiology, The First Affiliated Hospital of Nanjing Medical University and Jiangsu Province Hospital, Nanjing, Jiangsu, China; Bristol Heart Institute, University of Bristol, United Kingdom

## Abstract

During mouse heart development, ventricular endocardial cells give rise to the coronary arteries by angiogenesis. Myocardially-derived vascular endothelial growth factor-a (Vegfa) regulates embryonic coronary angiogenesis through vascular endothelial growth factor-receptor 2 (Vegfr2) expressed in the endocardium. In this study, we investigated the role of endocardially-produced soluble Vegfr1 (sVegfr1) in the coronary angiogenesis. We deleted sVegfr1 in the endocardium of the developing mouse heart and found that this deletion resulted in a precocious formation of coronary plexuses. Using an *ex vivo* coronary angiogenesis assay, we showed that the *Vegfr1*-null ventricular endocardial cells underwent excessive angiogenesis and generated extensive endothelial tubular networks. We also revealed by qPCR analysis that expression of genes involved in the Vegf-Notch pathway was augmented in the *Vegfr1*-null hearts. We further showed that inhibition of Notch signaling blocked the formation of coronary plexuses by the ventricular endocardial cells. These results establish that Vegfr1 produced in the endocardium negatively regulates embryonic coronary angiogenesis, possibly by limiting the Vegf-Notch signaling.

## Introduction

The embryonic heart consists of the endocardium, myocardium and epicardium. The endocardium is the inner epithelial cell layer of the heart and the epicardium is the outer epithelial layer; in between is the myocardium consisting of the cardiomyocytes. During heart development, the ventricular cardiomyocytes proliferate to form the compact myocardium and soon after, coronary plexuses develop within the myocardium. Coronary plexuses are the primitive coronary vessels, consisting of only the endothelium. These plexuses then fuse and recruit smooth muscle cells and fibroblasts to become the mature coronary arteries [1], [2], [3], [4], [5], [6].

The epicardium is derived from the proepicardium outside the embryonic heart [7], [8]. The progenitor cells within the epicardium differentiate into the coronary vascular smooth muscle cells through epithelial to mesenchymal transition [9], [10], [11], [12], [13], [14]. A subset of proepicardial cells also gives rise to coronary endothelial cells [15], [16], [17]. Different from the epicardium, the endocardium is derived from the vascular progenitor cells within the cardiogenic mesoderm [18], [19], [20], [21]. These progenitor cells undergo vasculogenesis to form an endocardial tube that separates the inner surface of the myocardium from the primitive heart chamber [22].

Endocardial cells specifically express nuclear factor in activated T-cell, cytoplasmic 1 (Nfatc1) during heart development [23], [24], [25], [26]. Our recent study in mice has shown that the *Nfatc1*-expressing endocardial cells give rise to the coronary arteries through angiogenesis via the molecular signaling from the myocardial vascular endothelial growth factor-a (Vegfa) to endocardial vascular endothelial growth factor receptor-2 (Vegfr2) [27]. Earlier studies in avian have also shown that the soluble vascular endothelial growth factor-receptor 1 (sVegfr1) blocks coronary vascular development [28]. sVegfr1 has no intracellular and transmembrane domain and negatively regulates the Vegfa signaling by competing with Vegfr2 for Vegfa during angiogenesis [29], [30], [31], [32], [33], [34]. Consistent with its inhibitory function for angiogenesis, global deletion of *Vegfr1* in mice results in endothelial overgrowth and disruptive primitive vessel formation [35]. However, global deletion of *Vegfr1* causes early embryonic death before coronary angiogenesis takes place and the potential role of sVegfr1 in this process in mice has not been studied.

In this study, we characterized the role of sVegfr1 in the embryonic coronary angiogenesis in mice by its genetic deletion in the endocardium using the *Nfatc1^Cre^*. We showed that such deletion resulted in premature coronary angiogenesis, leading to abnormal coronary plexuses, and augmented expression of endothelial genes, including *Dll4* in the Notch pathway. We also showed that inhibition of Notch signaling abated the coronary angiogenesis. These results confirm that sVegfr1 produced in the endocardium negatively regulate coronary angiogenesis and suggest that it limits the proangiogenic Vegf-Notch signaling in the ventricular endocardial cells while they undergo angiogenesis.

## Methods

### Mice

The floxed *Vegfr1* mice (*Vegfr1^f/f^*; a gift from Dr. Janet Rossant at University of Toronto and Dr. Kyunghee Choi at Washington University), floxed *R26^fsEGFP^* or *R26^fslacZ^* Cre reporter line [36], [37], and the *Nfatc1^Cre^* mice [27] were used in this study. They were maintained on the C57B6 background and genotyped by PCR using primers for *Vegfr1^f/f^* (CGCTTTTTGTCAGTCATCTTCA, GAGAATGCACTGTGCTGAAGGA), *R26^fsEGFP^* (CCCAAAGTCGCTCTGAGTTGTTATC, GAAGGAGCGGGAGAAATGGATATG), and *Nfatc1^Cre^* (GGCGCGGCAACACCATTTTT, TCCGGGCTGCCACGACCAA), respectively. Noontime on the day of observing vaginal plugs was designated as embryonic day (E) 0.5. All mouse experiments were performed according to the guideline of the National Institute of Health and approved by the Institutional Animal Care and Use Committee of Albert Einstein College of Medicine (IACUC number: 20110303).

### X-Gal Staining

Wholemount X-gal staining was performed as previously described [26]. E12.5 embryos were dissected, fixed in 4% PFA for 30 minutes at 4°C, washed twice in PBS containing 2 mM MgCl_2_ and once in PBS (pH 7.5)/2 mM MgCl_2_/0.1% deoxycholic acid/0.2% NP-40. The X-gal staining/reaction was developed in the same buffer containing 5 mM K3Fe(CN)6, 5 mM K4Fe(CN)6, and 0.6 mg/mL X-gal (Promega) at room temperature (RT) for 6 hours. The reaction was stopped by washing the embryos in PBS/0.5 mM EDTA. The stained embryos were photographed using a Zeiss SteREO Discovery V12 stereoscope. The stained embryos were then post-fixed, dehydrated, embedded in paraffin, sectioned and photographed using a Zeiss Axio Observer Z1 inverted microscope.

### Wholemount Pecam1 Immunostaining

Embryonic hearts were blocked in PBS containing 1% BSA and 0.1% Tween 20 and then incubated with a rat monoclonal anti-Pecam1 antibody (BD Biosciences) overnight at 4°C. After wash, the embryos were incubated with biotinylated anti-rat IgGs (Vector Labs). The color reaction was performed using the Elite Avidin-Biotin Complex (ABC) kit and DAB Peroxidase Substrate kit (Vector Labs). The stained hearts were photographed using the Zeiss SteREO Discovery V12 stereomicroscope. For each embryonic stage, 5 age-matched *Vegfr1^f/+^;Nfatc1^Cre^* (designated as control hereafter) and *Vegfr1^f/f^;Nfatc1^Cre^* embryos (designated as R1 CKO) were used for wholemount Pecam1 immunostaining.

### Sectional Immunohistochemistry

E11.5 whole embryos or E14.5 isolated hearts were fixed in 4% paraformaldehyde and processed for immunohistochemistry. Tissue sections were blocked in PBS containing 3% BSA and 0.05% Triton X-100 and incubated with rabbit polyclonal anti-Pecam1 antibodies (Santa Cruz Biotechnology, sc-1506) or a rabbit monoclonal anti-Cleaved Caspase3 antibody (Cell Signaling Technology, 9664). After wash, the sections were incubated with biotinylated anti-rabbit IgG (Vector Labs, BA-1000). The color reactions were developed using the ABC-AP and ABC-HRP for Pecam1 and Caspase3, respectively. Stained sections were photographed using the Zeiss Axio Observer Z1 inverted microscope. Five age-matched control and R1 CKO embryos or hearts were examined for immunochemistry.

### BrdU Incorporation and Cell Proliferation Assay

BrdU labeling reagent was intraperitoneally injected into the pregnant female mice 2 hours before the collection of E11.5 embryos. Tissue sections were prepared and immunostained using a BrdU Staining Kit (Invitrogen). The number of BrdU positive cells on 5 comparable ventricular sections from 3 age-matched control or R1 CKO embryos was counted and the data from the two groups were quantitatively analyzed and compared using the Student *t*-Test.

### In Vitro Coronary Angiogenesis Assay

The ventricles were dissected out from E11.5 control or *Vegfr1* null hearts by removal of the atrium, sinus, and outflow tract and placed into the growth factor reduced Matrigel (BD Biosciences, 356231) in the 4-well plates. The Matrigel was diluted 1∶1 with the M199 medium containing 2% fetal bovine serum and 10 ng/ml Vegf120 (R&D, 494-VE-005). Ventricular explants were cultured for 6 days and the angiogenesis by the EGFP-tagged endocardial cells was examined and photographed using a Zeiss SteREO Discovery V12 stereomicroscope. The number of angiogenic sprouts or endothelial pores produced by each cultured explant was quantitated and the data from control or R1 CKO ventricles (n  = 5 for each group) were analyzed using the Student *t*-Test.

### RNA Extraction and Quantitative PCR (qPCR)

Total RNA was isolated from pooled E11.5 ventricles of control or R1 CKO embryos using the Trizol® Reagent (Invitrogen) and reversely transcribed into cDNA using the SuperScript™ II Reverse Transcriptase (Invitrogen) and Oligo (dT)_18_ Primers (Fermentas Life Sciences). cDNAs from the two groups were added to the TaqMan® Array Plates and amplified using the StepOnePlus™ Real-Time PCR System (Applied Biosystems). Gapdh was used as a housekeeping gene for normalizing any loading differences. Stable expression level of Pecam1 was further used to normalize the number of endothelial cells for determination of any changes in the expression of endothelial genes per cell. Relative expression level was calculated by 2^-ΔΔCT^ method. Three individual pools of the ventricles from E11.5 control and R1 CKO embryos were used for qPCR analysis and the reactions were performed in triplicate for each sample.

### Statistical Analysis

Statistical analyses were carried out using the unpaired Student’s *t* test for analyzing difference in 2 groups or one-way ANOVA/Post Hoc Tukey’s test for analyzing difference within more than 2 groups. Values were present as means ± SD, and *p* value <0.05 was considered as statically significant for both tests.

## Results

### *Vegfr1* Produced in the Endocardium is Required for Normal Coronary Plexus Formation

**Figure 1:**
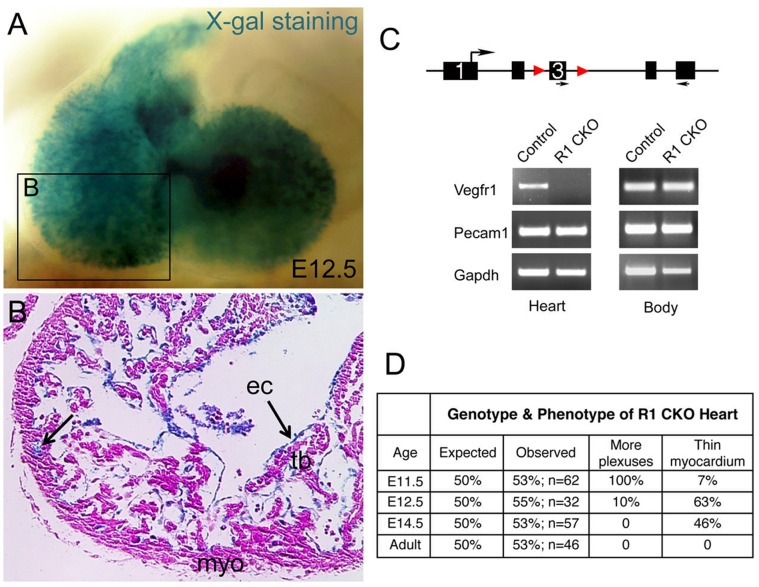
Tissue-specific knockout of *Vegfr1* in the endocardium by the *Nfatc1^Cre^* line. **A,** Wholemount X-gal staining of *R26^fslz^;Nfatc1^Cre^* E12.5 embryo showing the Cre-activated lacZ expression within the heart. **B,** A frontal sectional view of the cardiac chamber demonstrating lacZ expression in the endocardium (ec; arrows), but not in the compact myocardium (myo) or trabeculae (tb). **C,** Depicting the endocardial-specific deletion of Vegfr1 in the embryos by the *Nfatc1^Cre^* with RT-PCR analysis. **D,** A table summarizing the phenotypes of R1 CKO heart. An expected frequency (50%) of R1 CKO mice was found at different developmental stages and in adulthood, indicating that endocardial Vegfr1 was not required for survival. However, we observed a complete penetrance of early coronary plexus defect at E11.5, which only remained in a small subset of embryos at E12.5 and was not seen after E14.5. Additionally, half of E12.5 and E14.5 R1 CKO embryos had thin myocardium. The coronary phenotype was determined by immunohistochemistry, whereas the myocardial phenotype was determined by histology.

**Figure 2:**
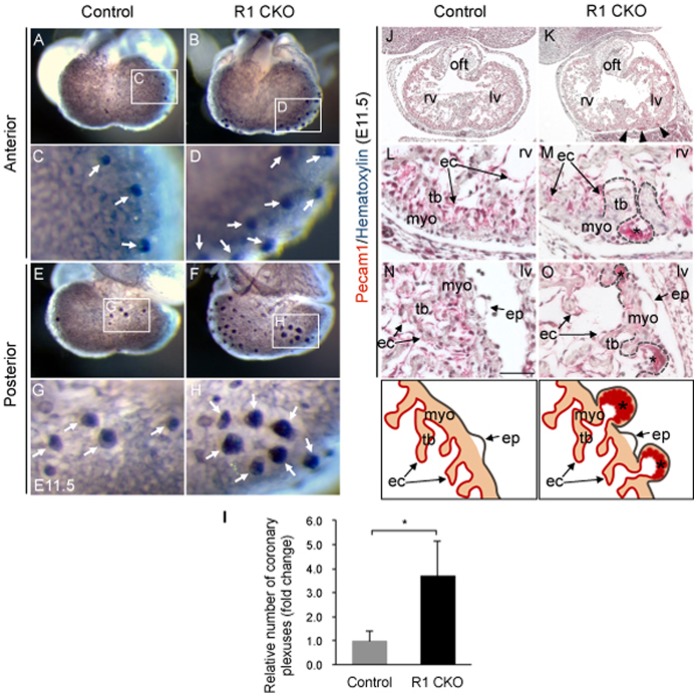
Disruption of *Vegfr1* in the endocardium results in the precocious and abnormal coronary plexuses. **A–H,** Wholemount Pecam1 immunostainings of E11.5 hearts show the newly formed coronary plexuses in the ventricles. Note greatly increased formation of these primitive vessels by the R1 CKO hearts (**B**, **D**, **F**, **H**) compared to the control hearts (**A**, **C**, **E**, **G**). **I,** Statistic analysis showing the increase is significant. n  = 7 hearts for each group, *p<0.05, error bars = SD. **J–O,** Immunostained frontal sections of E11.5 ventricles with Pecam1 antibodies showing that the precocious coronary plexuses (red staining) in the R1 CKO ventricles are dilated and resemble the primitive blood islands (**K**, **M**, **O**; indicated by arrowheads and asterisks), which are not present in the control hearts (**J**, **L**, **N**). Dash lines and schematic diagrams showing these early-formed abnormal coronary plexuses within the myocardium of the R1 CKO hearts. ep, epicardium; myo, myocardium; oft, outflow track; lv/rv, left/right ventricle; tb, trabeculae; scale bar  = 50 µm.

Embryonic coronary angiogenesis in mice takes place between E11.5 and E12.5. To determine the role of endocardially-produced Vegfr1 in this process, we disrupted *Vegfr1* in the endocardium by using the *Nfatc1^Cre^* and *Vegfr1^f/f^* mice. The Rosa26 Cre reporter line, *R26^fslacZ^* [37], was used to validate the endocardial-specific deletion, which showed the Cre-mediated lacZ expression present only in the endocardium of E12.5 heart (Fig. 1A, 1B). Further, semi-quantitative PCR analysis showed expression of sVegfr1 was completely abolished in the E11.5 R1 CKO hearts, whereas its expression outside the heart was not affected (Fig. 1C), thus confirming the tissue specificity and efficacy of sVegfr1 deletion. The deletion was not embryonic lethal; however, it resulted in abnormal primitive coronary vessels and thin myocardium (Fig. 1D). Specifically, wholemount Pecam1 staining revealed that the primitive coronary plexuses developed precociously in the R1 CKO hearts at E11.5 (Fig. 2A-H). Quantitative analysis confirmed that, while an average of 15 early vessel-forming sites was seen in the control heart at this stage, this number was significantly increased by 3-fold in the R1 CKO heart (Fig. 2I). Close examination of Pecam1-stainined sections showed that these precociously formed vessels by the *Vegfr1*-null endocardial cells were abnormally dilated; their lumens directly attached to the ventricular endocardium and penetrated through the ventricular myocardium into the subepicardial space (Fig. 2J, 2L, 2N vs. 2K, 2M, 2O).

### *Vegfr1* Regulates the Cell Fate of Coronary Plexuses

**Figure 3:**
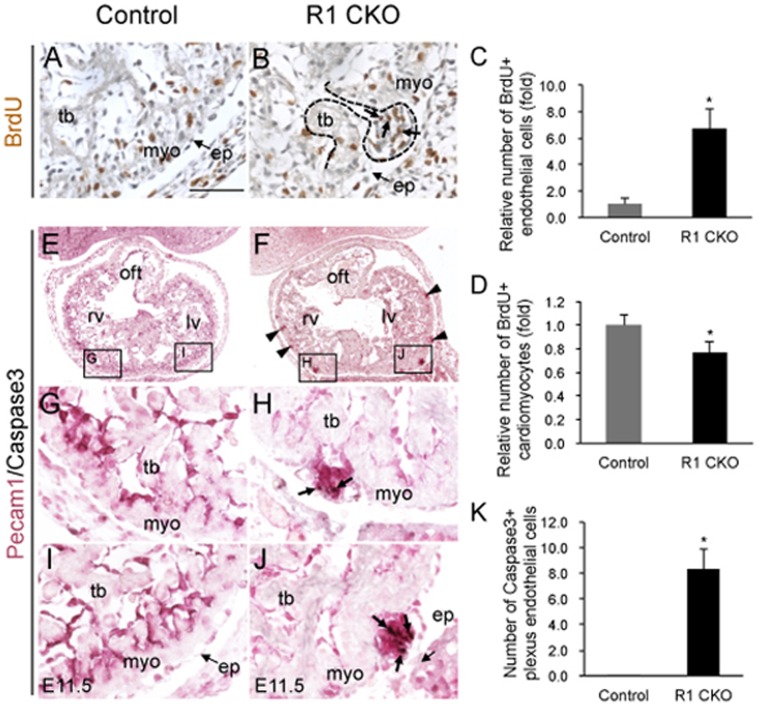
The coronary plexuses in the R1 CKO hearts are unstable and self-limited through apoptosis. **A, B,** Images of E11.5 ventricle immunostained with antibodies against BrdU (brown nuclear staining) showing the proliferating plexus endothelial cells in the R1 CKO hearts (**B**, indicated by arrows and dash line). No plexus is present in the same region of the control heart (**G**). Scale bar  = 50 µm. **C, D,** Statistical analyses showing a significantly increased proliferation of the plexus endothelial cells (**C**) and decreased proliferation of cardiomyocytes (**D**) in the E11.5 R1 CKO hearts. n  = 3 individual hearts, 5 comparable sections per heart, error bars = SD. **E-J,** Images of the frontal sections of E11.5 ventricles co-immunostained with the antibodies against Pecam1 (purple membrane staining) and Caspase3 (black nuclear staining) showing the apoptotic endothelial cells within the overgrowing coronary plexuses in the R1 CKO embryos (**F**, arrowheads; **H** and **J**, arrows). No apoptosis is present in the control hearts (**E**, **G**, **I**). **K,** Quantitative analysis showing a significantly increased apoptosis of the R1 CKO endothelial cells. n  = 3 individual hearts, 5 comparable sections per heart, error bars = SD.

To determine if these precocious coronary vessels were associated with over-proliferation of endothelial cells, we purse-chased E11.5 embryos for 2 hours with BrdU and harvested the hearts for co-immunostaining of BrdU and Pecam1. The staining revealed that while the numbers of the BrdU-positive endocardial cells were comparable between the control and CKO hearts (Fig. 3A vs. 3B), the endothelial cells of the immature vessels derived from the *Vegfr1*-null endocardial cells were mostly BrdU-positive, forming highly proliferative coronary plexuses (Fig. 3B, 3C). In contrast, quantitative analysis also showed a 23% decrease in the number of BrdU-positive cardiomyocytes in the R1 CKO hearts (Fig. 3D). We also co-immunostained the embryonic hearts with Caspase3 and Pecam1 antibodies. The results showed that a significant portion of plexus endothelial cells in the E11.5 R1 CKO hearts was positive for Caspase3 (Fig. 3F, 3H, 3J, 3K). These observations suggest that the increased coronary plexuses in the E11.5 R1 CKO hearts are formed by overproliferation, yet these plexuses are not stable and undergo apoptosis.

**Figure 4:**
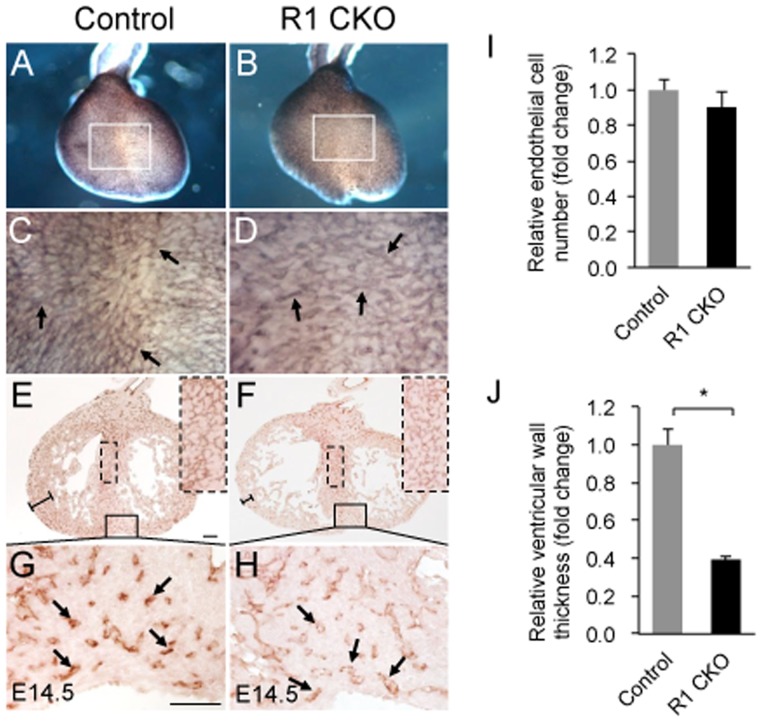
Endocardial *Vegfr1* is not essential for late coronary development, but required for normal ventricular wall development. A-D, Images of wholemount Pecam1 stained E14.5 hearts showing comparable coronary vasculatures (arrows indicating individual vessels) between the control and R1 CKO hearts. E-H, Images of Pecam1 stained frontal sections of E14.5 hearts showing comparable coronary vasculatures between the control and R1 CKO hearts (arrows indicating individual vessels). Note that the CKO hearts have a thin compact myocardium. Scale bar  = 50 µm. I, Quantitative analysis showing comparable numbers of coronary endothelial cells between E14.5 control and R1 CKO hearts. n  = 3 individual hearts, 5 comparable sections per heart, error bars = SD. J, Quantitative analysis showing that the thickness of the compact myocardium is significantly reduced in the R1 CKO embryos compared to the control embryo. n  = 3 individual hearts; 5 comparable sections per heart. *p<0.05, error bars = SD.

We next examined E14.5 hearts by Pecam1 wholemount and sectional staining and found that the coronary networks at this later stage were comparable between the control (Fig. 4A, 4C, 4E, 4G) and R1 CKO hearts (Fig. 3B, 3D, 3F, 3H). Quantitative analysis confirmed that the numbers of endothelial cells were similar between the two groups (Fig. 4I). Despite the recovery of the coronary vascular development from its early defect, the ventricular wall thickness of the R1 CKO hearts was significantly reduced (Fig. 4E, 4F, 4J).

**Figure 5:**
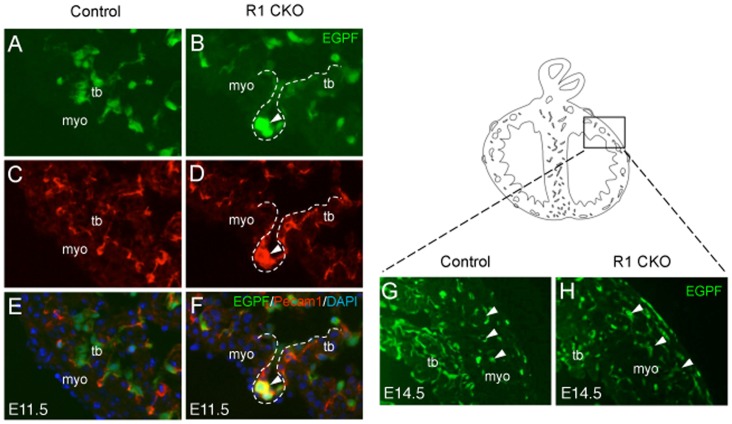
R1 CKO endocardial cells generate the precocious coronary plexuses. **A, B,** Image of cultured E11.5 ventricle showing EGFP labeled plexus (**B,** arrowhead). **C, D,** Images showing Pecam1 stained plexus (**D,** arrowhead). **E, F,** Merged images showing EGFP/Pecam1 co-staining plexus (**F,** arrowhead). **G, H,** EGFP-labeling showing comparable coronary vasculatures between the E14.5 control (**G**) and R1 CKO heart (**H**). myo, myocardium; tb, trabeculae.

To determine whether the precocious plexuses were derived from the R1 CKO ventricular endocardial cells, we performed the cell fate-mapping analysis in the triple transgenic *Nfatc1^Cre^;R26^fsEGFP^;Vegfr1^f/f^* embryos. The result showed that the precocious plexuses in the E11.5 R1 CKO hearts were formed by the EGFP-tagged and Pecam1-positive cells (Fig. 5A-F), thus confirmed that these immature coronary plexuses were originated from the R1 CKO endocardial cells. The fate-mapping analysis also revealed comparable coronary vasculatures formed between the control and R1 CKO hearts at E14.5 (Fig. 5G, 5H). Together, the results from these cell fate studies suggest that the endocardially-produced Vegfr1 may play two independent roles in the coronary angiogenesis and ventricular morphogenesis. In addition, the early-formed coronary plexuses in the R1 CKO hearts are likely self-eliminated through apoptosis in the later coronary development. This may explain why the early precocious coronary formation does not result in persistent coronary vascular defects.

### Endocardial *Vegfr1* Negatively Regulates Coronary Angiogenesis

**Figure 6:**
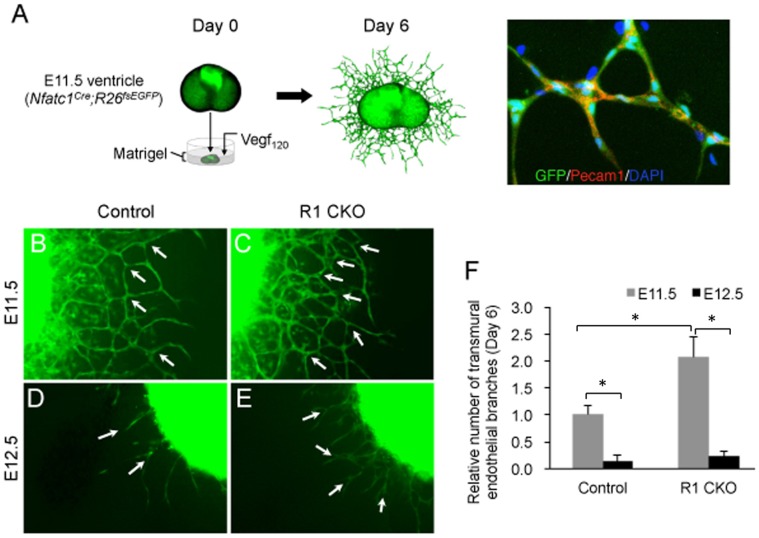
R1 CKO endocardial cells form excessive coronary plexuses by augmented coronary angiogenesis. **A,** Schematic diagram and EGFP/Pecam1 double labeling illustrating that Vegf_120_ promotes angiogenesis by the genetically labeled ventricular endocardial cells in the Matrigel culture of E11.5 *Nfatc1^Cre^;R26^fsEGFP^* ventricle to form endothelial tubular networks. **B, C,** Images of endothelial networks developed from the cultured ventricles of the E11.5 *Nfatc1^Cre^;R26^fsEGFP^* (control) (**B**) or *Nfatc1^Cre^;R26^fsEGFP^;Vegfr1^f/f^* (R1 CKO) embryos (**C**) showing excessive endothelial tube formation (arrows) by the R1 CKO endocardial cells. **D**, **E,** Images of E12.5 ventricular explants showing that endothelial tube formation by the endocardial cells are greatly reduced in both control (**D**) and R1 CKO (**E**) hearts, although R1 CKO ventricles still form more endothelial tubes. **F,** Statistical analysis showing that angiogenic branching occurs mainly at E11.5 and the process is reduced significantly at E12.5. Of note, there is significantly increased endothelial branching by the R1 CKO endocardial cells at E11.5. n  = 5 ventricular explants per group, *p<0.01, error bars = SD.

To reveal if Vegfr1 also negatively regulated the angiogenic process, we performed an *ex vivo* embryonic coronary angiogenesis assay. In this assay, we isolated the ventricles from the E11.5 *Nfatc1^Cre^;R26^fsEGFP^* (control) or *Nfatc1^Cre^;R26^fsEGFP^;Vegfr1^f/f^* (R1 CKO) embryos and cultured them in the matrigel. We used Vegf_120_ to induce the angiogenesis by the ventricular endocardial cells and visualized their angiogenic movement by EGFP, including invasion of the ventricular wall and subsequently formation of endothelial networks confirmed by their expression of Pecam1 (Fig. 6A). Using this approach, we showed that the angiogenesis by the endocardial cells occurred mostly at E11.5 (Fig. 6B, 6C) and the process subsided by E12.5 in either group (Fig. 6B, 6D), the R1 CKO cells formed increased vascular networks (Fig. 6D, 6E). Quantitative analysis confirmed that the R1 CKO endocardial cells generated significantly more endothelial branches compared to the control cells at E11.5; the difference was diminished at E12.5 when the angiogenic process was greatly weakened (Fig. 6F). These results support that sVegfr1 inhibits the Vegfa-induced Vegfr2 signaling in the endocardial cells for the onset of coronary angiogenesis [27]; removal of Vegfr1 results in the premature coronary angiogenesis by these cells. The data also reveal a narrow developmental window for the Vegf signaling and Vegfr1 action on the initiation of coronary vessel formation.

### *Vegfr1* Limits the Vegf-Notch Signaling Required for Coronary Angiogenesis

**Figure 7:**
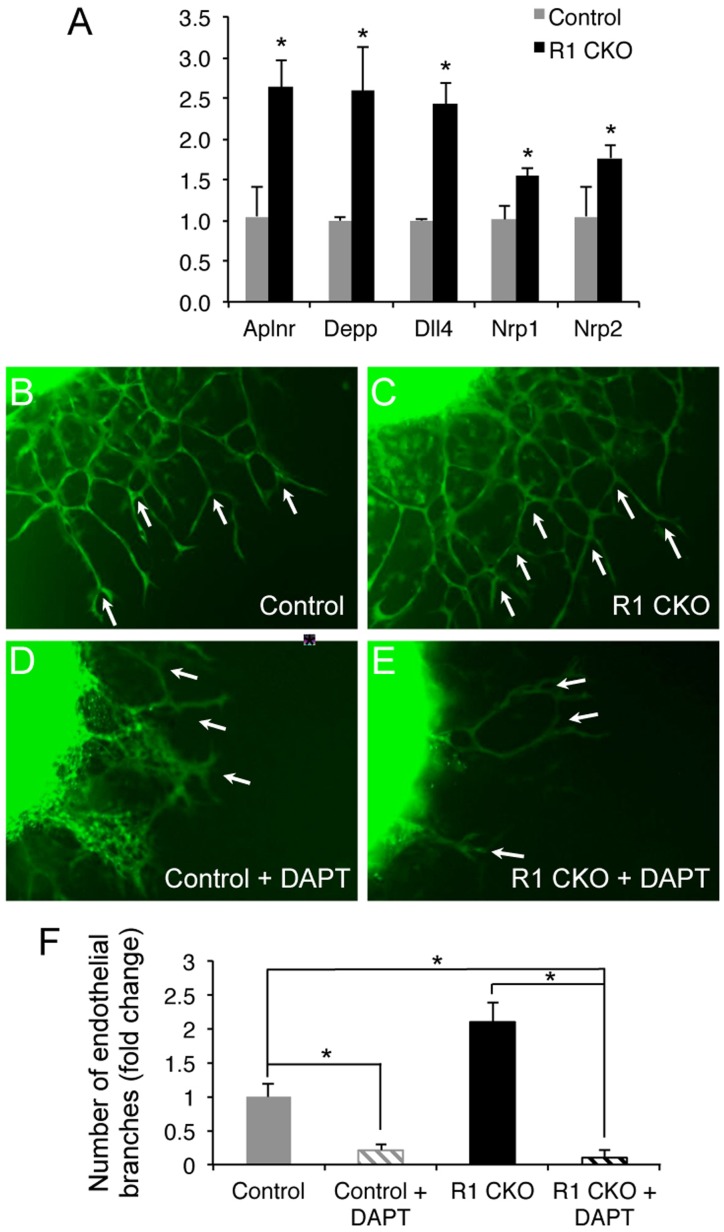
Notch signaling mediates Vegf_120_-induced coronary angiogenesis by the ventricular endocardial cells. **A.** A graph showing increased expression of 5 angiogenic/vascular endothelial genes in the E11.5 R1 CKO hearts. **B-E,** Images of E11.5 ventricular explants showing that Notch signaling inhibitor DAPT abolished the Vegf120-induced angiogenic branching (arrows) by the control (**B**, **D**) and R1 CKO endocardial cells (**C**, **E**). **F,** Statistical analysis showing that DAPT treatment significantly inhibits the endothelial branching by the control or R1 CKO endocardial cells. n  = 5 ventricles, *p<0.05, error bars = SD.

To explore the mechanisms by which Vegfr1 inhibits the coronary angiogenesis, we examined expression of angiogenic or endothelial genes in the E11.5 control and R1 CKO ventricles using qRT-PCR and found that, among 23 genes examined (Table S1), expressions of Aplnr, Depp, Dll4, Nrp1, and Nrp2 were significantly increased in the R1 CKO ventricles (Fig. 7A). Of note, Dll4 functions in the Notch pathway and interact with Vegf signaling to regulate vascular endothelial differentiation and angiogenesis [38], [39], [40], [41]. To determine whether Notch signaling is responsible for the increased embryonic coronary angiogenesis by the R1 CKO endocardial cells, we blocked the Notch signaling in the *ex vivo* coronary angiogenesis assay by using DAPT [41]. We found that DAPT treatment abolished the Vegf_120_-induced coronary angiogenesis by either by either control or R1 CKO endocardial cells (Fig. 7B-F). Taken together, these results suggest that Notch signaling is essential for the Vegfa-induced coronary angiogenesis and suggest that Vegfr1 suppresses the Vegf-Notch signaling in the endocardial cells thereby limiting their angiogenic differentiation during the coronary angiogenesis.

## Discussion

**Figure 8:**
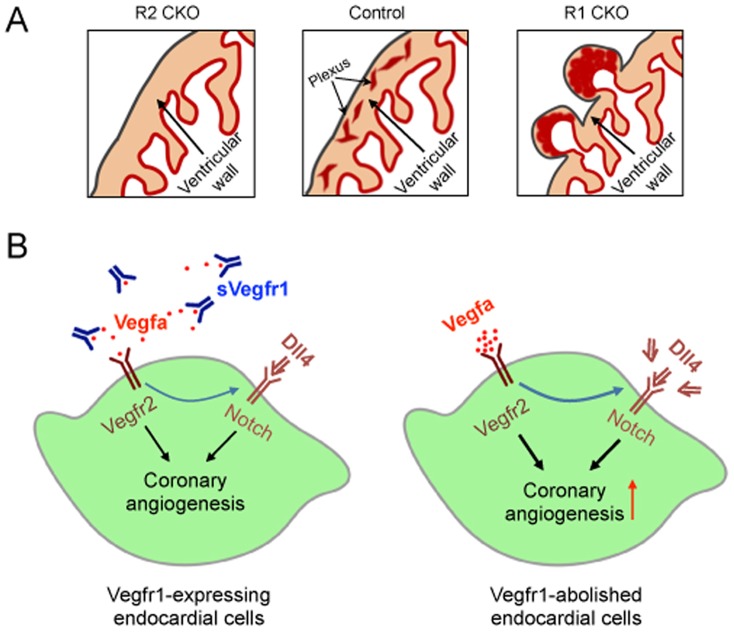
Working model shows the Vegf-Notch signaling in the ventricular endocardium that regulates coronary angiogenesis by the ventricular endocardial cells. A, Schematics showing that a balanced Vegf signaling in the endocardium (Control) is essential for the coronary angiogenesis by the ventricular endocardial cells. When the Vegf signaling in the endocardium is disrupted by deleting the proangiogenic Vegfr2 (R2 CKO) [27] or the anti-angiogenic Vegfr1 (R1 CKO; this study), the coronary angiogenesis is blocked or accelerated, respectively. B, Diagrams depicting a mode of controlled Vegf-Notch signaling that is necessary for normal coronary angiogenesis. Enhanced Vegf2 signaling in the endocardial cells by removal of the inhibitory sVegfr1 results in the increased Dll4 expression and accelerated augmented coronary angiogenesis whereas blocking Notch signaling prohibits the process (not shown in the diagrams), suggesting that balanced Vegf and Notch signals collaborate in the endocardial cells to control the coronary angiogenesis.

We have recently shown that the myocardial Vegfa to endocardial Vegfr2 signaling is essential for embryonic coronary angiogenesis by the progenitor cells within the endocardium to form the coronary arteries [33]. In this study using a conditional deletion strategy, we show that the endocardially-derived Vegfr1 is also required for normal coronary angiogenesis (Fig. 8A), it negatively regulates the process, possibly limiting the proangiogenic Vegf-Notch signaling.

Vegfr1 is a negative regulator of Vegf signaling through its soluble form, sVegfr1, which has no intracellular and transmembrane domain [29], [30], [31], [32], [33], [34]. It binds Vegfa, Vegfb, and Pigf. In mice, sVegfr1 acts as a decoy receptor of Vegfr2 and by binding to Vegfa, it suppresses the major proangiogenic signaling of Vegfa to Vegfr2. Global deletion of Vegfr1 in mice results in early embryonic death due to endothelial overgrowth and disruptive primitive vessel formation [35]. In quails, injection of sVegfr1 into the embryonic hearts inhibits the formation of coronary plexuses likely through binding to Vegfb [28], [42].

The cardiac endocardial cells and vascular endothelial cells have the same embryonic origin and share most of the cellular makers and functions [43], [44]. Like in the vascular endothelium where loss of sVegfr1 causes peripheral vascular defects, including the overgrowth of endothelial cells and disorganized vascular pattern [32], [33], [35], our *in vivo* deletion study shows that loss of sVegfr1 in the cardiac endocardium results in excessive formation of abnormal coronary plexuses and overexpression of endothelial genes including Dll4. Further *ex vivo* coronary angiogenesis assay reveals increased angiogenesis as a major underlying mechanism of the defect. Thus, the current study establishes a tissue-specific role of Vegfr1 in the endocardium required for coronary vessel formation.

Vascular angiogenesis requires angiogenic sprouting from a selected subpopulation of endothelial cells [45], [46], [47]. Interaction of reciprocal Vegf and Notch signaling coordinates this selection [48]. Specifically, Dll4, a transmembrane ligand in the Notch pathway, expressed by angiogenic cells, activates Notch signaling in adjacent stalk endothelial cells to suppress Vegf activities and limits endothelial sprouting [38], [49], [50]. In parallel, sVegfr1 released from the stalk endothelial cells acts on the neighboring angiogenic cells to guide their directional sprouting [32]. We show in this study that loss of Vegfr1 in the endocardium upregulates expression of Dll4 during coronary angiogenesis and Notch signaling is necessary for the process. This observation suggests that Vegf and Notch signalings collaborate in the endocardial cells to select a subset of endocardial cells for coronary angiogenesis (Fig. 8B).

Another noticeable finding of this study is that, unlike the embryos with the pan-vascular endothelial deletion of *Vegfr1* that die in early development, the embryos with the endocardial deletion sustain the earlier coronary defect and are survived to birth. We do not know the mechanism for the later recovery, though it may be due to the apoptosis of the overgrown *Vegfr1*-null endothelial cells. It is also not known from our analysis that whether the augmented Notch signaling is involved in the death of plexus cells. Future study is required to understand how Vegfr1 regulates Vegf-Notch signaling in the endocardium to control the embryonic coronary angiogenesis.

## Supporting Information

Table S1 List of endothelial gene expression examined by qRT-PCR.
(DOCX)
