# Supplementary material for: Accelerated Coronary Angiogenesis by Vegfr1-Knockout Endocardial Cells
Source: PLoS One. 2013 Jul 24;8(7):e70570. doi: 10.1371/journal.pone.0070570 (PMC3722158; doi:10.1371/journal.pone.0070570)
Supplement: Table S1 — List of endothelial gene expression examined by qRT-PCR. (DOCX) [file pone.0070570.s001.docx]

| **Pan-endothelium** | **Arterial endothelium** | **Venous endothelium** |
| --- | --- | --- |
| Cdh5, Pecam1, Tie1, Tie2, Vwf | Depp, Dll4,Efnb2, Foxc1, Foxc2, Hes1, Hey1, Hey2, Jag1, Jag2, Notch1, Notch4, Nrp1 | Aplnr, Dll1, Ephb4, Nr2f2, Nrp2 |

**Table S1. List of vascular endothelial genes examined by qRT-PCR**
